# Early examination of real-world uptake and second-dose completion of recombinant zoster vaccine in the United States from October 2017 to September 2019

**DOI:** 10.1080/21645515.2021.1879579

**Published:** 2021-04-13

**Authors:** Brandon J. Patterson, Chi-Chang Chen, Catherine B. McGuiness, Lisa I. Glasser, Kainan Sun, Philip O. Buck

**Affiliations:** a US Health Outcomes & Epidemiology, GSK, Philadelphia, PA, USA [Employment at Initial Submission]; b IQVIA, Pennsylvania, PA, USA

**Keywords:** Herpes zoster, shingles, postherpetic neuralgia, recombinant zoster vaccine, vaccine, immunization, public health

## Abstract

*Shingrix* (Recombinant zoster vaccine, RZV) was approved in October 2017 in the United States (US) for the prevention of herpes zoster in adults aged 50 years and older. The vaccine is administered in two doses, with the second dose administration recommended between two and six months after the first dose. Examination of uptake and series completion is important to ensure appropriate use, especially at the time of vaccine introduction. This report provides demographic characteristics of patients receiving RZV between October 2017 and September 2019, first- and second-dose uptake, and a cumulative estimation of second-dose completion by month for US adults aged 50 years and older. Monthly uptake increased rapidly since October 2017; overall, 7,097,441 first doses of RZV were administered along with 4,277,636 second doses during the observed timeframe. Among people with an observed first-dose administration, 70% and 80% completed the two-dose series within six and 12 months post initial dose, respectively. This evidence suggests that RZV has rapidly been adopted by a large population in the US and most are following manufacturer or policy recommendations regarding series completion. Further analyses are needed to explore potential patient, provider, and policy-relevant characteristics associated with second-dose completion that could serve as targets for further improvement.

## Introduction

Herpes zoster (HZ), also referred to as shingles, occurs upon reactivation of latent varicella zoster virus (VZV). Primary infection with VZV leads to chickenpox and it is estimated that in the United States (US) more than 90% of the population will be infected before adolescence.^1^ After infection, VZV remains dormant in the cranial, dorsal root, sacral, and autonomic ganglia. With increasing age, the risk of developing HZ increases due to a decline in cell-mediated immunity or immunosuppression;^2^ people living up to 85 years have a 50% chance of suffering an episode of HZ during their lifetime.^3^

HZ is characterized by a unilateral, vesicular rash, typically affecting one dermatome and causing intense pain.^4^ Even though the initial rash usually resolves within two to four weeks, costly complications such as postherpetic neuralgia (PHN) may develop.^5,6^ PHN is defined as pain persisting at least 90 days after resolution of the HZ rash. PHN-related pain is often described as “itching, stabbing or burning” sensation and may persist for several months to years, thereby affecting day-to-day functioning of patients and reducing their quality of life.^4,7,8^ Antivirals and painkillers are the cornerstone of HZ therapy, but there is no clinical evidence that the use of antivirals prevents development of PHN.^9^ Pharmacological treatment of PHN-related pain includes tricyclic antidepressants, gabapentinoids and opioids in patients refractory to first- and second-line treatment options.^10^ However, patient satisfaction with treatment remains low,^11,12^ highlighting the complexity and difficulty of treating PHN-related pain.^10^

Vaccination against HZ with either live-attenuated zoster vaccine or adjuvanted recombinant zoster vaccine (RZV) increases cellular and humoral immune responses in people, thereby providing protection against HZ and PHN.^13,14^ In people aged 50 years and older, RZV vaccination reduced HZ-related burden of pain by >90% compared with placebo.^8^ RZV was approved by the Food and Drug Administration in October 2017 and has since received preferential recommendation by the Advisory Committee on Immunization Practices (ACIP) in adults aged 50 years and older for prevention of HZ.^15^ RZV should be administered as two doses, separated by two to six months in order to achieve optimal immunogenicity and efficacy as demonstrated in two large phase III studies and post-marketing studies.^16–19^ Post-hoc analyses of limited phase III data showed reduced vaccine efficacy in subjects receiving only one RZV dose, i.e., 90.8% and 69.5% in adults ≥50 years and ≥70 years, respectively, compared with estimated vaccine efficacies of 97.2% and 91.3% in these age groups.^18,20^ In a post-marketing phase III study, the humoral immune response of two RZV doses given six and 12 months apart was compared to RZV doses given two months apart. Vaccine response rates one month after the second RZV dose were >90% for all dosing schedules, but the 0, 12 months dosing schedule did not meet the non-inferiority criterion compared with the 0, two months dosing schedule.^17^ Nevertheless, the ACIP recommended not to restart the dosing series if more than six months had elapsed after the first dose; thus, some people might receive the two doses at longer intervals.^15^ Published cost-effectiveness models have assumed different completion rates of the two-dose vaccine course varying from 56% to 100%,^15,21,22^ yet there is little information regarding real-world second-dose completion with RZV in the US. An accurate description of completion rates and factors associated with RZV dosing schedule compliance may help in designing strategies to increase completion and compliance and optimizing protection against HZ. Therefore, the objective of this study was to assess cumulative vaccine uptake, second-dose completion levels, and adherence to dosing recommendations in the US using large, geographically dispersed, and representative datasets.

## Methods

This was a retrospective study utilizing IQVIA’s open source longitudinal prescription claims and medical claims datasets. The longitudinal prescription claims data are derived from electronic information received from pharmacies, payers, software providers, and transactional clearinghouses and represent activities that take place during a prescription transaction. The medical claims data include pre-adjudicated claims from more than 870,000 practitioners per month from a wide set of practices. These claims datasets have a significant national footprint and include data from all payers: cash, commercial, Medicare, and Medicaid. Furthermore, these data are updated more rapidly than other datasets, as data are nearly complete within one to two months.

US adults aged 50 years and older receiving an initial dose of RZV between October 2017 and September 2019 were identified and followed. Exclusions were made for data quality issues (e.g., missing age, gender, etc.) and receipt of a second dose within 31 days after the initial dose, but no other criteria relating to required observation time were applied. Receipt of RZV doses were identified using NDC 58160-819-12, 58160-829-01, 58160-828-01, 58160-823-11, 58160-829-03, 58160-828-03, or CPT code 90750. Cumulative completion rate at pre-defined time points (i.e., two, three, six, nine, or 12 months post initial dose) and corresponding 95% confidence intervals were calculated using Kaplan-Meier methods by censoring patients at the time of second-dose vaccination or at the end of follow-up, whichever was earlier. Demographic characteristics of subjects at time of initial dose were described.

## Results

**Figure 1.**
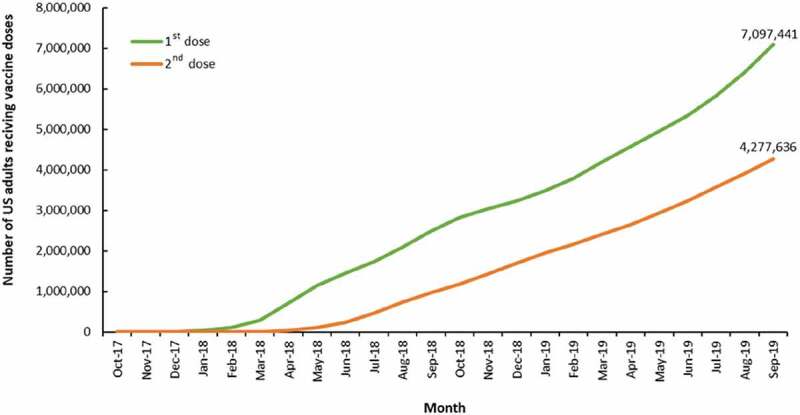
Cumulative number of first and second doses of RZV administered from October 2017 through September 2019

Of the 7,167,310 US adults aged 50 years and older who had a claim consistent with an initial dose of RZV in the datasets, 7,097,441 were included in further analyses after application of the exclusion criteria. Figure 1 illustrates a linear increase in first-dose RZV administrations starting from March 2018 with subsequent second-dose RZV administrations beginning in June 2018 and steadily increasing to 4,277,636 second doses administered by September 2019.

RZV: recombinant zoster vaccine

Demographic information about the subjects at the time of initial dose are presented in Table 1. The mean and median age of subjects at initial dose was 68.49 and 68.00 years, respectively. The largest proportion (32.3%) of subjects received the initial dose when aged 70-79 years. Most subjects (57.9%) were females and lived in the South (34.7%), as expected since the South is the most populated region in the US. Half of the initial dose claims (50.1%) were paid by non-Medicare commercial payers and the majority (91.6%) were processed as a prescription benefit claims.

**Table 1.** Demographic characteristics at time of initial RZV dose (N = 7,097,441)

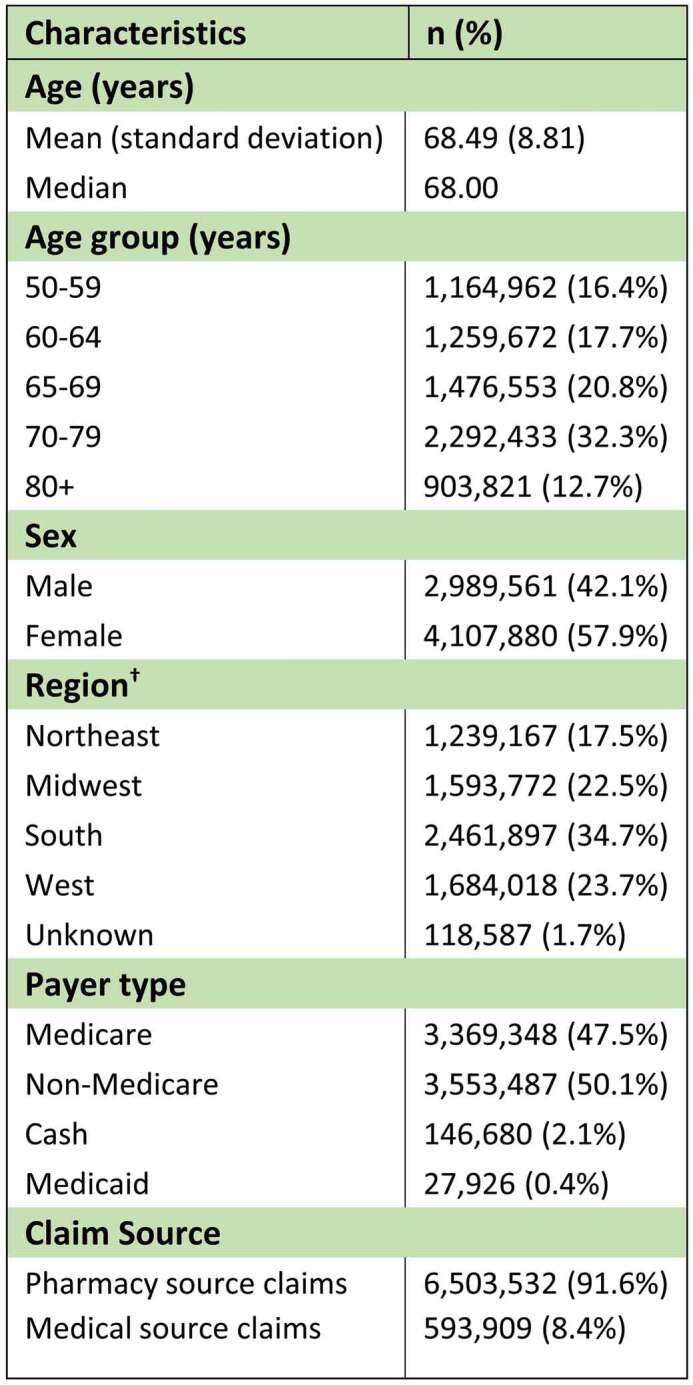

†Census regions and Divisions are available at https://www2.census.gov/geo/pdfs/maps-data/maps/reference/us_regdiv.pdf

n: number of patients; RZV: recombinant zoster vaccine

Cumulative rates of RZV second-dose completion according to monthly uptake are shown in Table 2. The shading of the table indicates when subjects would have enough potential observable time to allow for complete observation of the time period specified in the columns. Averages of RZV second-dose completion are estimated at 3% within 60 days, 36% within 90 days, 70% within six months, 77% within nine months, and 80% within 12 months post initial dose in those with sufficient observation time.

**Table 2.** Cumulative proportion of people with second-dose completion

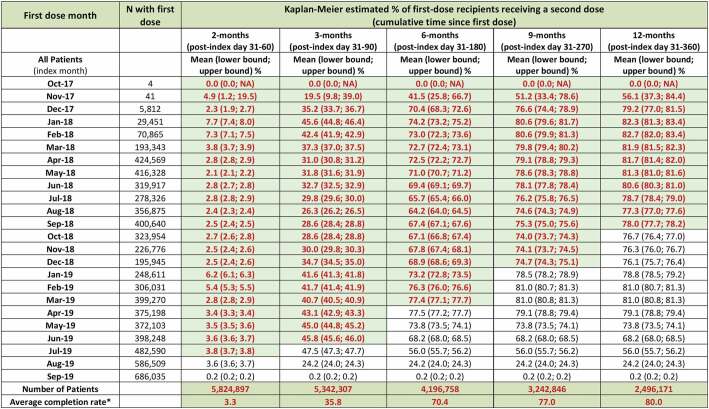

**Notes**: Index month corresponds to month of first RZV prescription. Shaded cells represent second-dose rates (and corresponding 95% confidence interval bounds) during the time period when patients in each month/row (first-dose month) had the possibility to contribute to second-dose data based on the follow-up period. For example, patients who received their first dose in Jan-19 could possibly be followed up for 8 months (i.e., Feb-19 through Sep-19) before the end of the observable time in the datasets (i.e., Sep-19).

*Weighted average based on shaded-cell data (completion rate and N) and not the entire sample.

CI: confidence interval; N: number of patients; NA: not applicable; RZV: recombinant zoster vaccine

## Discussion

This publication reports initial uptake and estimates of RZV second-dose completion in US adults aged 50 years and older using extensive, nationally distributed representative datasets.

An analysis of real-world data, presented to the ACIP, indicated that uptake of RZV increased rapidly since its approval in October 2017 and compliance was >70% and >80% within six and nine months after the initial dose, respectively.^23^ Our results are in line with these data: estimated second-dose completion rates were 70 and 80% within six and 12 months post initial dose, respectively, for those with adequate observation time.

We found that second-dose completion exceeds that for other vaccine series used in adults and was similar to that reported for RZV in Canada (65.0% and 74.9% for completion rates within six and 12 months post initial dose, respectively).^24^ Retrospective studies have shown that completion rates of Hepatitis A and B vaccine series were typically low, ranging between 25% and 65% depending on the vaccine, age and payer type.^25,26^ However, the target population for Hepatitis A and B (adults ≥19 years with additional risk factors or another indication) differs substantially from the target population of RZV,^27^ which may explain the observed differences in completion rates. On the other hand, observed RZV US completion levels were below those seen for pediatric vaccines.^26^Among healthcare providers, fear of side effects and/or needles and lack of insurance coverage were cited as potential hurdles for starting and completing recommended immunization schedules.^28^ In this regard, reactogenicity experienced with RZV administration was not inconsequential in pivotal trials and could potentially impact second-dose completion.^29,30^ Results from clinical trials suggested that the overall reactogenicity profiles after first and second RZV dose are comparable but that there is limited correlation between the intensity of RZV-related adverse events experienced by individual patients after first and second RZV dose.^29,30^ In a post-hoc analysis of clinical phase III studies, the frequency of a given grade 3 adverse event after the second RZV dose was highest among people who already experienced the same grade 3 adverse event after the first dose.^29^ Reactogenicity and impact on the quality of life was further evaluated in an open-label, single-arm study including 401 adults. Grade 3 reactogenicity events occurred in 9.5% of subjects with a transient impact on quality of life, which typically resolved in one to two days.^30^ Overall, quality of life was not affected by a single RZV dose for most people. This information might be helpful for heathcare professionals to manage expectations, inform patients of potential side effects and measure these against the benefit of RZV vaccination in order to increase compliance. Indeed, the present study appears to suggest robust completion levels but does not allow for evaluating whether adverse events have played into a person’s decision to forego the second RZV dose. Another reason for suboptimal completion or a > six months interval between first and second RZV dose could be a shortage in vaccine supply.^31^A combination of increased awareness of HZ among older adults, the publicized high vaccine efficacy of RZV and the rapid endorsement by ACIP may have led to a higher than anticipated demand, leading to temporary shortage of RZV.^31^ As for zoster vaccine live, the first HZ vaccine on the market, recommendation by health care professionals and creation of vaccine opportunities could increase HZ vaccine uptake.^32^

Limitations of the study include comprehensiveness of records and applicability beyond the dataset. While it may not be the entirety of RZV doses administered in the US since its launch, a great majority were captured in the study. These data may not be generalizable to other populations where unmeasured factors could influence RZV completion rates, nor may it be representative of future utilization patterns. Unforeseen exogenous circumstances such as supply shortages and pandemics may negatively influence uptake and completion of the RZV series. However, the stable proportion of people completing the two-dose RZV series over time demonstrated in this study would suggest the potential to overcome transient negative effects.

In conclusion, this report describes rapid uptake and high proportion of second-dose completion of RZV series among US adults aged 50 years and older. Relative stability over time of second-dose prescription claims was also shown. Additional studies will be needed to elucidate patient, provider, and payer variables associated with completion levels, and to identify important subpopulations and other factors most in need of intervention by providers and policymakers. The higher than anticipated uptake may be due to increased awareness of the debilitating pain associated with HZ and the preferential recommendation provided by ACIP. Coverage by commercial and public health insurers may have lowered financial hurdles, leading to increased demand. Observations regarding RZV uptake suggest that patient education and recommendations issued by governmental bodies and healthcare providers may boost vaccine uptake and compliance in older adults, which is of particular importance in light of the current pandemic.

## Abbreviations

ACIP: Advisory Committee on Immunization Practices
HZ: Herpes zoster
VZV: Varicella zoster virus
RZV: Recombinant zoster vaccine
PHN: Postherpetic neuralgia
US: United States

## Trademark

*Shingrix* is a trademark owned by or licensed to the GSK group of companies.

## References

[1] Gershon AA, Breuer J, Cohen JI, Cohrs RJ, Gershon MD, Gilden D, Grose C, Hambleton S, Kennedy PG, Oxman MN, et al. Varicella zoster virus infection. Nat Rev Dis Primers. 2015;1:15016. doi:10.1038/nrdp.2015.16. 27188665 PMC5381807

[2] Mueller NH, Gilden DH, Cohrs RJ, Mahalingam R, Nagel MA. Varicella zoster virus infection: clinical features, molecular pathogenesis of disease, and latency. Neurol Clin. 2008;26:675–97. doi:10.1016/j.ncl.2008.03.011. 18657721 PMC2754837

[3] Cohen JI. Clinical practice: herpes zoster. New England J Med. 2013;369:255–63. doi:10.1056/NEJMcp1302674. 23863052 PMC4789101

[4] Oxman MN. Herpes zoster pathogenesis and cell-mediated immunity and immunosenescence. J Am Osteopath Assoc. 2009;109:S13–S17. 19553630

[5] Patterson BJ, Rausch DA, Irwin DE, Liang M, Yan S, Yawn BP. Analysis of vascular event risk after herpes zoster from 2007 to 2014 US insurance claims data. Mayo Clin Proc. 2019;94:763–75. doi:10.1016/j.mayocp.2018.12.025. 30955916

[6] Yawn BP, Saddier P, Wollan PC, St Sauver JL, Kurland MJ, Sy LS. A population-based study of the incidence and complication rates of herpes zoster before zoster vaccine introduction. Mayo Clin Proc. 2007;82:1341–49. doi:10.4065/82.11.1341. 17976353

[7] Drolet M, Brisson M, Schmader KE, Levin MJ, Johnson R, Oxman MN, Patrick D, Blanchette C, Mansi JA. The impact of herpes zoster and postherpetic neuralgia on health-related quality of life: a prospective study. CMAJ. 2010;182:1731–36. doi:10.1503/cmaj.091711. 20921251 PMC2972323

[8] Curran D, Oostvogels L, Heineman T, Matthews S, McElhaney J, McNeil S, Diez-Domingo J, Lal H, Andrews C, Athan E, et al. Quality of life impact of an adjuvanted recombinant zoster vaccine in adults aged 50 years and older. J Gerontol A Biol Sci Med Sci. 2019;74:1231–38. doi:10.1093/gerona/gly150. 29955836 PMC6625590

[9] Chen N, Li Q, Yang J, Zhou M, Zhou D, He L. Antiviral treatment for preventing postherpetic neuralgia. Cochrane Database Syst Rev. 2014;2:CD006866. doi:10.1002/14651858.CD006866.pub3. 24500927 PMC10583132

[10] Argoff CE, Katz N, Backonja M. Treatment of postherpetic neuralgia: a review of therapeutic options. J Pain Symptom Manage. 2004;28:396–411. doi:10.1016/j.jpainsymman.2004.01.014. 15471658

[11] Gater A, Abetz-Webb L, Carroll S, Mannan A, Serpell M, Johnson R. Burden of herpes zoster in the UK: findings from the zoster quality of life (ZQOL) study. BMC Infect Dis. 2014;14:402. doi:10.1186/1471-2334-14-402. 25038799 PMC4223600

[12] Oster G, Harding G, Dukes E, Edelsberg J, Cleary PD. Pain, medication use, and health-related quality of life in older persons with postherpetic neuralgia: results from a population-based survey. J Pain. 2005;6:356–63. doi:10.1016/j.jpain.2005.01.359. 15943957

[13] Chlibek R, Pauksens K, Rombo L, van Rijckevorsel G, Richardus JH, Plassmann G, Schwarz TF, Catteau G, Lal H, Heineman TC. Long-term immunogenicity and safety of an investigational herpes zoster subunit vaccine in older adults. Vaccine. 2016;34:863–68. doi:10.1016/j.vaccine.2015.09.073. 26432913

[14] van der Heiden M, de Rond LGH, van Zelm MC, Berbers GAM, Boots AMH, Buisman AM. Age-dependent pre-vaccination immunity affects the immunogenicity of varicella zoster vaccination in middle-aged adults. Front Immunol. 2018;9:46. doi:10.3389/fimmu.2018.00046. 29410671 PMC5787056

[15] Dooling KL, Guo A, Patel M, Lee GM, Moore K, Belongia EA, Harpaz R. Recommendations of the advisory committee on immunization practices for use of Herpes Zoster vaccines. Morb Mortal Wkly Rep. 2018;67:103–08. doi:10.15585/mmwr.mm6703a5. PMC5812314 29370152

[16] Hastie A, Catteau G, Enemuo A, Mrkvan T, Salaun B, Volpe S, Smetana J, Rombo L, Schwarz T, Pauksens K, et al. Immunogenicity of the adjuvanted recombinant zoster vaccine: persistence and anamnestic response to additional doses administered 10 years after primary vaccination. J Infect Dis. 2020:jiaa300. doi:10.1093/infdis/jiaa300. PMC8672743 32502272

[17] Lal H, Poder A, Campora L, Geeraerts B, Oostvogels L, Vanden Abeele C, Heineman TC. Immunogenicity, reactogenicity and safety of 2 doses of an adjuvanted herpes zoster subunit vaccine administered 2, 6 or 12 months apart in older adults: results of a phase III, randomized, open-label, multicenter study. Vaccine. 2018;36:148–54. doi:10.1016/j.vaccine.2017.11.019. 29174683

[18] Lal H, Cunningham AL, Godeaux O, Chlibek R, Diez-Domingo J, Hwang SJ, Levin MJ, McElhaney JE, Poder A, Puig-Barberà J, et al. Efficacy of an adjuvanted herpes zoster subunit vaccine in older adults. N Engl J Med. 2015;372:2087–96. doi:10.1056/NEJMoa1501184. 25916341

[19] Cunningham AL, Lal H, Kovac M, Chlibek R, Hwang SJ, Diez-Domingo J, Godeaux O, Levin MJ, McElhaney JE, Puig-Barberà J, et al. Efficacy of the Herpes Zoster subunit vaccine in adults 70 years of age or older. N Engl J Med. 2016;375:1019–32. doi:10.1056/NEJMoa1603800. 27626517

[20] Curran D, Van Oorschot D, Varghese L, Oostvogels L, Mrkvan T, Colindres R, et al. Assessment of the potential public health impact of Herpes Zoster vaccination in Germany. Hum Vaccin Immunother 2017; 13:2213–2221. 28708959 10.1080/21645515.2017.1345399 PMC5647993

[21] Curran D, Patterson B, Varghese L, Van Oorschot D, Buck P, Carrico J, et al. Cost-effectiveness of an Adjuvanted Recombinant Zoster Vaccine in older adults in the United States. Vaccine 2018; 36:5037–5045. 30017145 10.1016/j.vaccine.2018.07.005

[22] Le P, Rothberg MB. Cost-effectiveness of the adjuvanted Herpes Zoster subunit vaccine in older adults. JAMA Intern Med. 2018;178:248–258. doi:10.1001/jamainternmed.2017.7431. 29297049 PMC5838796

[23] Dooling KL, HZ Work Group Liaison. Herpes zoster vaccines: update. ACIP meeting Herpes Zoster Vaccine; 2019.

[24] McGirr A, Bourgoin T, Wortzman M, Millson B, McNeil S. An early look at the second dose completion of the recombinant zoster vaccine (RZV) in Canadian adults – a retrospective analysis. Canadian Immunization Research Network; 2019. 10.1016/j.vaccine.2021.04.053 34001346

[25] Ghaswalla PK, Patterson BJ, Cheng WY, Duchesneau E, Macheca M, Duh MS. Hepatitis A, B, and A/B vaccination series completion among US adults: a claims-based analysis. Hum Vaccin Immunother. 2018;14:2780–85. doi:10.1080/21645515.2018.1489189. 29923789 PMC6314407

[26] Nelson JC, Bittner RC, Bounds L, Zhao S, Baggs J, Donahue JG, et al. Compliance with multiple-dose vaccine schedules among older children, adolescents, and adults: results from a vaccine safety datalink study. Am J Public Health. 2009;99(Suppl 2):S389–97. 19797753 10.2105/AJPH.2008.151332 PMC4504385

[27] Centers for Disease Control and Prevention (CDC). Recommended adult immunization schedule for ages 19 years or older, United States, 2020. CDC, ed. 2020.

[28] Johnson DR, Nichol KL, Lipczynski K. Barriers to adult immunization. Am J Med. 2008;121:S28–S35. doi:10.1016/j.amjmed.2008.05.005. 18589065

[29] Colindres R, Wascotte V, Brecx A, Clarke C, Hervé C, Kim JH, Levin MJ, Oostvogels L, Zahaf T, Schuind A, et al. Post hoc analysis of reactogenicity trends between dose 1 and dose 2 of the adjuvanted recombinant zoster vaccine in two parallel randomized trials. Hum Vaccin Immunother. 2020:1–6. 32347767 10.1080/21645515.2020.1741312 PMC7733973

[30] Schmader KE, Levin MJ, Grupping K, Matthews S, Butuk D, Chen M, et al. The Impact of reactogenicity after the first dose of recombinant zoster vaccine on the physical functioning and quality of life of older adults: an open-label, Phase III Trial. J Gerontol Ser A Biol Sci Med Sci. 2019;74:1217–24. 30256905 10.1093/gerona/gly218 PMC6625580

[31] McGee P.Uptake of herpes zoster vaccine linked to supply.In: Internist A, ed. Infectious Disease; 2019.

[32] Williams WW, Lu PJ, O’Halloran A, O’Halloran A, Bridges CB, Kim DK, et al. Vaccination coverage among adults, excluding influenza vaccination - United States, 2013. Morb Mortal Wkly Rep. 2015;64:95–102. PMC4584858 25654611

